# Human resource management at the intensive care unit: A pragmatic review and future research agenda for building a learning health system

**DOI:** 10.1002/lrh2.10395

**Published:** 2023-10-18

**Authors:** Wim J. R. Rietdijk, P. Hugo M. van der Kuy, Corstiaan A. den Uil

**Affiliations:** ^1^ Department of Hospital Pharmacy Erasmus University Medical Center Rotterdam The Netherlands; ^2^ Department of Institutional Affairs Vrije Universiteit Amsterdam Amsterdam The Netherlands; ^3^ Department of Intensive Care Maasstad Hospital Rotterdam The Netherlands

**Keywords:** burnout, human resources management, intensive care units, management, organizational theory, work engagement

## Abstract

Recently, the importance of efficient and effective health care has been recognized, especially during the acute phase of the Coronavirus Disease‐2019 (COVID‐19) pandemic. Intensive care units (ICUs) have faced an immense workload, with massive numbers of patients being treated in a very short period of time. In general, ICUs are required to deliver high‐quality care at all times during the year. At the same time, high‐quality organizational goals may not be aligned with the interests, motivation, and development of individual staff members (eg, nurses, and doctors). For management of the ICU, it is important to balance the organizational goals and development of the staff members (“their human capital”), usually referred to as human resource management. Although many studies have considered this area, no holistic view of the topic has been presented. Such a holistic view may help leadership and/or other stakeholders at the ICU to design a better learning health system. This pragmatic review aims to provide a conceptual model for the management of ICUs. Future research may also use this conceptual model for studying important factors for designing and understanding human resources in an ICU.

**Abbreviations**
HRM: human resource management
ICU: intensive care unit
JD‐R model: job demand‐resources model
LHS: learning health system

## 1 INTRODUCTION

In recent years, the importance of efficient and effective health care has been recognized, especially during the Coronavirus Disease‐2019 (COVID‐19) pandemic, when intensive care units (ICUs) faced an immense workload with the massive numbers of patients to treat in a very short period of time.^1^, ^2^, ^3^, ^4^ On the one hand, ICUs are required to deliver high‐quality care at all times during the year.^5^, ^6^, ^7^, ^8^, ^9^, ^10^, ^11^ At the same time, such high‐quality organizational goals may not be aligned with the interests, motivation, and development of individual staff members (ie, support staff, nurses, and doctors). For management of the ICU, it is of great importance to balance the organizational goals and work engagement and development of the staff members. Ultimately, the staff working in the ICU are the *human capital* of the organization, as typified recently in a study on how to build organizational and individual resilience in the ICU.^12^ In organizational theory, this is referred to as human resource management (HRM).

Recent research in the critical care literature has examined aspects related to the work of nurses and doctors in health care^13^, ^14^, ^15^, ^16^, ^17^, ^18^, ^19^ and intensive care in particular.^12^, ^20^, ^21^, ^22^, ^23^, ^24^, ^25^, ^26^, ^27^, ^28^ Especially related to burnout,^24^, ^29^, ^30^, ^31^ work engagement,^25^, ^26^ conflict resolution within teams,^32^ and other aspects related to the perception of working at the ICU.^14^, ^15^, ^16^ This shows the importance of the topic, but a more holistic view of the topic is missing. This is particularly relevant as societal pressures (eg, digitization and use of artificial intelligence and shortage of personnel) require to design and manage a learning health system. Of course, understanding of the prevalence and factors influencing of burnout or the characteristics of work engagement is an important part. However, for the leadership of a department, a holistic view of the HRM topics and practices and the design of future work is essential.

For these reasons, our pragmatic review surveys the present HRM and intensive care literature, this with the aim to build a learning health system (LHS). In one of the foundational studies on this topic,^33^ five features of a LHS were identified. These ranged from individual patient data, to implementing the best practices and attempting to continuously improve processes. This is in line with sociotechnical structures and the interest of stakeholders. In order to contribute, as an health care department, to these LHS features, health care workers (eg, nurses, doctors) are an important group to manage to achieve the underlying goals of an LHS. Especially in the time of societal developments and pressures, an LHS approach is essential to strive for excellent care with the limited resources. For this purpose, we provide a conceptual model of relevant factors taken from the human resources (HR) literature that may provide an overview for staff working in the ICU.

This pragmatic review is structured as follows. First, the important dimensions of the conceptual model are outlined. Second, the main important factors in the conceptual model are discussed with the latest developments of these topics. Finally, possible future studies are discussed as well as the implications for the management of an ICU. It is important to note that the present review is not an exhaustive list of all the literature, but it merely presents a comprehensive overview of the main topics that may be of interest to intensive care staff.

## 2 HRM: EXISTING FRAMEWORKS AND CONCEPTUAL MODEL DEVELOPMENT

This pragmatic review builds upon major topics related to the design and execution of work, focusing on particular topics of interest to the ICU. Four major themes are identified: personality and motivation; interpersonal processes; group, department, and organization; and external factors that impact HRs of the organization.^14^, ^16^

Before going into the critical care setting, we need an introduction to general models and developments within the HRM field. An early HRM study describes both the hard and soft versions of HRM. Where the hard versions of HRM focus on management control systems and an economic approach to managing the workforce^34^; the soft controls are more taking into account experiences of employees.^34^ Intuitively, and LHS should use a multilevel approach. This entails management control systems that are initiated from the management team of a department but it should also take into account the interest of the daily workforce (eg, nurses and doctors).^35^

**FIGURE 1:**
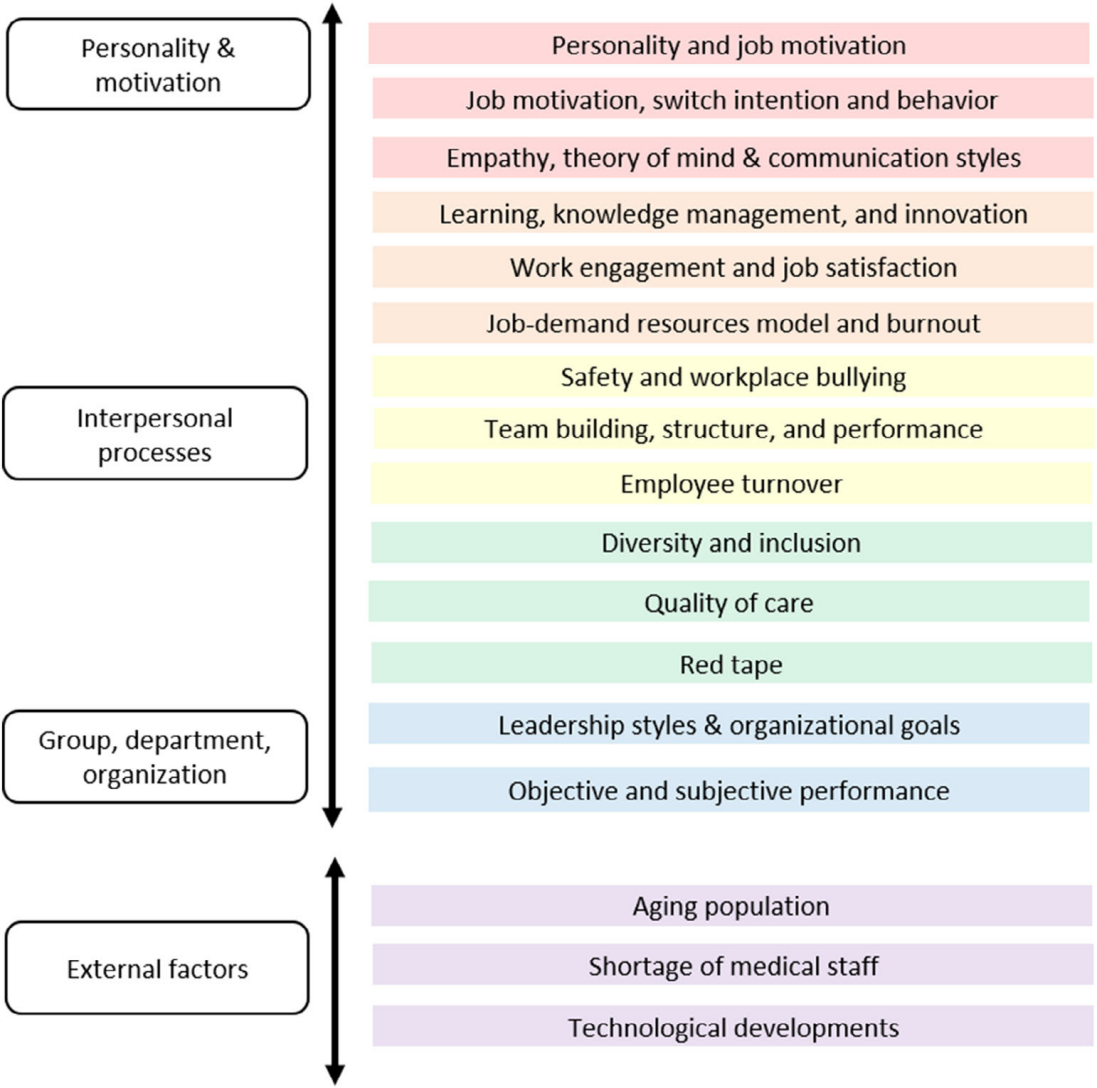
Conceptual model of human resource management topics relevant to the intensive care unit.

Here, we define HRM as an approach to effectively and efficiently manage people in an organization, focusing on policies and control systems.^36^ The overall purpose of HRM is to ensure that the organization is able to achieve success through the staff.^37^ HRM can be employed at several levels of the organization, ranging from an individual staff member to the board room of a hospital. Therefore, we would like to describe major HRM topics related to these four themes. Figure 1 presents a conceptual model of the topics discussed in this pragmatic review.

## 3 PERSONALITY AND JOB MOTIVATION

As a start, the individual staff member brings along their own personality, experience, and motivation to the ICU. At work, showing motivation to perform is an essential part of work and designing and managing HR policies. Individuals constitute a team, and a team is important for performing tasks that are relevant to the ICU. For this reason, this pragmatic review starts with an overview of important factors that are related to individuals working in the ICU.

### 3.1 Personality

Personality refers to the differences in patterns in how individuals think, feel, and behave.^38^ Scientifically, we would refer to them as cognitive, affective, and behavioral processes, respectively. Generally, research into these individual differences focuses on particular aspects (eg, empathic ability or resiliency) or the interplay between these aspects.^38^ For example, studies examine how work engagement interacts with empathic ability when attempting to explain job performance? Additionally, a well known, frequently used personality test concerns the Big Five personality model. Research has uncovered the five dimensions in personality (ie, neuroticism, extraversion, openness, conscientiousness, and agreeableness) that are found to be quite stable over time.^39^

Research has focused on the association between personality and job performance (eg, Refs. 40, 41). In particular, the Big Five personality traits are used to explain job performance. Although the relation is context dependent,^41^ the Big Five might have some degree of utility for selecting employees into a variety of jobs. In particular, conscientiousness is one of the most valid predictors of performance for most jobs (see Ref. 41). At the ICU, being aware of individual differences, especially the Big Five, is an important first step in understanding how someone's personality plays a role in their performance at work.

### 3.2 Job motivation

Job motivation is described as the combination of several “energetic forces” within an employee and the interaction of the employee and the job environment to get the job done.^42^ Job motivation has played a central role in studying performance in organizational theory since the early 1960s.^42^, ^43^ In contemporary work, motivation research integrates all sorts of factors related to the individual's personality, cognition, emotions, work environment, and behavior.^42^, ^43^ Especially higher vitality, resilience and support are thought to have a positive impact on motivation to perform a job.^25^, ^26^ However, the actual results are still to be published.^26^ However, it is well known that a higher work motivation positively impacts productivity on the job.^44^

### 3.3 Job switching intention and behavior

While on the job, an individual staff member may become unsatisfied with the job, need a new environment, or seek a better opportunity elsewhere (eg, may get a higher salary in another job). In turn, these factors lead to the job switch intention. Either of the three major reasons for switching intentions lead to a lower motivation at work, and eventually leads to switching behavior.^45^, ^46^ A body of literature has examined these drivers and processes of job switching behavior,^10^, ^45^, ^46^, ^47^, ^48^, ^49^, ^50^, ^51^ particularly in a hospital setting.^46^

## 4 INTERPERSONAL PROCESSES

Interpersonal processes are cognitive, affective, and behavioral processes that take pace in the interaction between the individual staff member, colleagues, and department. Several models play a role in the ICU, for example, the extent to which nurses and doctors empathize with each other and the patients. What communication styles are important in interacting with people at work? Furthermore, formal processes are highlighted, such as learning, knowledge management, and innovation. Additionally, models of work engagement in relation to job satisfaction and performance are touched upon. Finally, a major theme at work is burnout. In several major studies, the relationship between the work engagement model and burnout prevalence has been shown. The section *interpersonal processes* conclude with a discussion of perceived safety and workplace bullying.

### 4.1 Empathy, theory of mind, and communication styles

Intensive care, and health care in general, is concerned with two important interpersonal processes: empathy and communication. Empathy is an important process that helps to understand and feel the emotions of others.^52^ At the ICU, this process helps doctors and nurses to interact with patients. Recent studies have shown that empathy increases a patients' trust, compliance, and satisfaction.^53^, ^54^, ^55^ Although empathy is a multifaceted construct, we think theory of mind would fit better as compared to empathy as a theory.^22^ Where, empathy generally refers to an affective mind relating and tuning into the emotions of others,^53^ theory of mind concerns merely the “distant” observation of the state of mind of others.^56^ In medicine, a thin line exists between cognitively and affectively showing empathy and still keep professional distance to the patient. Nevertheless, these interpersonal processes are immensely important in caring for a patient.

Furthermore, communication styles have important consequences at work in health care.^57^, ^58^, ^59^, ^60^, ^61^ Communication in the intensive care happens in meetings, patient rounds, spontaneous conversations and may happen synchronous as well as asynchronous.^57^ Synchronous happens when participants are there at the same time in the same meeting, while messages on white boards may also be seen as signs of asynchronous communication. Hall^58^ states that health care is a complex environment, where nurses and doctors should have the ability to communicate with patients and a more broad audience of stakeholders. The importance of effective communication is underlined in several reviews, especially with patient quality of care and outcomes.^57^, ^59^, ^61^ However, more research is needed to design an effective educational intervention that guides effective communication with colleagues and patients in health care. Such designs may be borrowed from other professional disciplines that have proven to be effective, such as with mediation techniques.^62^

### 4.2 Learning, knowledge management, and innovation

In health care, developments in treatments are rapidly evolving.^63^, ^64^ Learning new skills, treatments, and ways of working are required to keep up with these developments.^65^ At a managerial level, intensive care leaders should be aware of knowledge management practices and theories.^66^ This to enhance access to these developments, create a learning environment for their personnel and manage knowledge as an asset.^66^ In today's economy, digitization is changing health care systems, although information technology adoption is generally slow.^67^ For example, e‐consults (ie, consults between health care provider and patient using telecommunication) are a new mode by which physicians may interact with their patients and family. In addition, telemedicine and distant monitoring of ICU patients is performed.^68^, ^69^, ^70^ All these examples require a different mindset of medical staff, as physical examinations of the patients are impossible.

Together with learning new skills and updating old ones, knowledge goes hand in hand with innovation.^71^, ^72^, ^73^ Roberts and colleagues introduce the notion of design thinking by which innovation can be managed in a health care setting.^74^, ^75^, ^76^ Design thinking is human‐centered research approach where there is an interactive collaboration in diverse teamwork, where the aim is to rapidly prototype new ideas, processes, and products.^74^ Similar to this line of thinking and as an example of such innovation in the ICU, thoughts were initiated about designing the *silent ICU*.^77^ It was thought that by optimally designing the cacophony of IV and monitoring sounds patients and medical staff perception of quality of care may be improved,^77^ yet studies about this topic in the ICU are still to be performed.

### 4.3 Work engagement and job satisfaction

A long‐standing organizational psychology model that has been studied is work engagement and job satisfaction. Kahn^78^ introduced the concept of work engagement and defined this as the way employees are actively involved in their works (ie, either physically, cognitively, and emotionally) and that they perceive that they perform well in their jobs. Suggestively, employees who are engaged invest more attention in their work and performance.^79^ This theory and factor has been recognized by other researchers as an important factor in organizational psychology.^25^, ^79^, ^80^, ^81^, ^82^

Moreover, there is the concept of job satisfaction. Early on, Locke^83^ described job satisfaction as the pleasure an employee has with performing the content of the work. Recent research showed a positive correlation between work engagement and job satisfaction.^25^, ^26^, ^84^ In turn, both work engagement and job satisfaction result in a higher retention rate. This suggests that engagement and job satisfaction both may contribute to higher well‐being at work,^81^, ^82^ also for health care workers^17^ and in the ICU in particular.^25^, ^26^, ^85^

### 4.4 The job‐demand resource model and burnout

A seminal theory in recent decades is the job‐demand resources (JD‐R) model.^80^, ^86^ This theory examines various characteristics of the job, burnout that may explain job performance.^86^ An assumption of the JD‐R model is that there are two broad categories for defining job characteristics as an explanatory factor for job performance: job demands and job resources. *Job demands* refer to all required aspects of work, which an employee should possess in order to perform the job. These aspects are related to the physical, psychological, and organizational needs that usually have “costs” to the employee.^86^ Usually, these aspects are related to the more negative aspects of a job. Examples of job demands are high work and emotional pressure due to for example interaction with patients. *Job resources* refer to the same aspects of work that are instrumental in achieving the organizational or departmental goals. In addition, these Job resources stimulate an individual's development and may also contribute to the lower job demands (in line with^86^). In general, job resources are interpreted as the more positive aspects of the job. Examples include the perception that an employee has a secure job, an employee has the chances to develop oneself and receives a proper salary.

Disengagement from work of employees occurs when there are extended periods of emotional, mental, or physical pressure at work,^80^, ^87^ and ultimately may lead to burnout.^80^, ^88^ In turn, burnout is a central theme that has been studied in health care^18^, ^89^ and in the ICU, in particular.^24^, ^25^, ^26^, ^29^, ^87^ In addition, recent studies showed the importance of the JD‐R model and its aspects are important for explaining job performance of nursing staff.^14^, ^15^, ^16^, ^90^ The ICU ward and staff faced enormous pressure during the COVID‐19 pandemic.^26^ This resulted in high stress levels, high physical pressure, and emotional pressure. Consequently, many nurses disengaged from or even left work. Therefore, this should be a central theme for designing HR policies at the ICU.

### 4.5 Safety and workplace bullying

Work should be a place where colleagues feel safe to perform their job. Neal and Griffen^91^ developed a model to study workplace safety. They state that a safe work climate contributes to a professional interaction between staff members and even has a positive impact on job performance.^91^ Especially in the ICU context, this is an important factor, as it may impact the quality of care and patient safety.^92^, ^93^

When staff members experience an unsafe work climate, one of the factors that may contribute to this is workplace bullying. Workplace bullying is an understudied but important topic. As in any department, politics, and so‐called “undercurrents” occur. Sometimes, underlying emotions and thoughts about staff members are not expressed for a variety of reasons (eg, colleagues feel unsafe speaking out). Such “undercurrents” may be driven by the different ambitions and interests of staff members or are related to future career choices and options. This “competition” among colleagues jeopardizes a healthy work atmosphere and even leads to bullying behavior. In a recent study, Al Omar et al.^92^ found that workplace bullying behavior negatively impacts the quality of care and patient safety.

## 5 TEAM, DEPARTMENT, AND ORGANIZATION

The next section focuses on the team, department, and organization. This includes how teams are formed and structured to the extent to which red tape (“bureaucracy”) plays a role in leadership and objective and subjective performance and goals.

### 5.1 Team building and structure

A key topic in the health policy and management literature is team building,^94^ although not much team‐based research has been performed to date.^19^, ^94^, ^95^ Additionally, few interventions have addressed enhancing team building among health care teams,^95^ and those published suffer from biased groups, lack of control conditions, and inconsistent outcome measures.

Although an important question remains, how to grow and establish effective clinical teams^6^? In a recent study, it was found that the quality of teams is improved using a more formal design of policies. Such policies should be directed at aligning department/organizational goals with the personal interest of the individual team members and the interaction between team members.^6^ In addition, other topics that contribute to effective clinical teams are related to effective communication styles (as discussed previously),^60^ clear decision‐making processes, perceived workplace safety, and the ability to resolve potential issues and conflicts.^6^ A key element of effective team building, as suggested by previous research, is related to the “colleague's strengths recognition.”^96^ The ability to see the strengths in someone's work and personality, and use these strengths as complements to one own's.^96^ However, more research is needed into how this could be done effectively in ICU teams. A final factor that we would like to discuss in effective team building is leadership. In a later paragraph, we will revisit and discuss leadership styles.

### 5.2 Employee turnover at the department and organizational level

Job switching intention and behavior have been discussed above. Usually, poor engagement and low job satisfaction are reasons for employees having the intention of switching and actual switching behavior.^47^, ^84^ On a departmental level using a managerial perspective, employee turnover is important for two reasons.^97^ First, a high employee turnover rate may be a signal that the work environment is neither motivating or engaging.^84^, ^97^, ^98^ Second, a high employee turnover rate is very costly.^97^, ^99^ When employees switch jobs quickly, the cost of recruitment, hiring, and on‐boarding (eg, getting the employees ready to work) is high. This is especially true in knowledge‐intensive industries, such as the ICU. Additionally, forced employee turnover, for example, due to a restructuring or reorganization of departments, may result in low work engagement and satisfaction and consequently high turnover (intentions).

### 5.3 Diversity and inclusion

Relatively recently, diversity and inclusion have been made an explicit item on the agenda of many management and administrative bodies,^100^, ^101^, ^102^ where there is an overrepresentation of white individuals in many medical specialties.^103^ Diversity and inclusion are not only limited to ethnicity, sex, and/or gender but may also include personality, neurodiversity, sexual orientation, and religious beliefs. A central—and dominant—paradigm in diversity and inclusion includes intersectionality. Intersectionality refers to the diversity within a person (how does an individual identifies in demographic dimensions such as gender, ethnicity, religious beliefs, etc.) and how this diversity relates to or interacts with the work environment.^104^ To increase diversity at work,^104^ an intersectional approach is relevant. Such an approach captures the changing realities of for example family and workforce. Examples of such changing realities are family compositions, work–life balance discussions, and changing current social norms (eg, only celebrating Christian holidays in Western Europe and no parental leave for fathers). The diversity and inclusion of the health care workforce are not only important for representation within organizations, but may also provide a good signal to patients admitted to the ICU. Mostly, patients bring a background when admitted to the ICU that—when the doctor may sympathize better with them as they are also from a more diverse background—results in a stronger trusting relationship.

Finally, diversity and inclusion also play a role in ICU research. Recently, women's leadership in research is promoted.^105^ The *Journal of Critical Care* dedicated an special issue to publishing studies with only first and senior female authors. These initiatives are applauded for, as these steps are important to change the status quo—that eventually shifts to a more representative (academic) workforce.

### 5.4 Red tape

Public sector organizational theory is concerned with studying the extent to which red tape exists and influences work perception.^106^, ^107^, ^108^ Red tape is generally considered from an individual's perspective, rather than that there are objective measures for it (in line with^109^). Red tape is defined as (perceived) excessive pressure for employees to comply to guidelines and procedures that may not necessarily have an effect in proper decision‐making and management of a primary process.^109^ Further, Kaufman^108^ describes it as the senseless rules within the organization and the fact that there is no pressure to comply to these rules. It is well known that this phenomenon has a major impact on perception, work engagement, satisfaction, and performance (eg Ref. 110), although studies into red tape in health care are limited in number.^111^ Although red tape is generally an individual's perception on excessive administrative duties—we argue—it may also include actual behavior. For example in health care, there is a major pressure to measure all sorts of aspects of patients multiple times during a day. While, many of these documented data will not change much over the course of the day. This may result in excessive pressure to document the patient's status and may distract from the key aspects of the care job of a nurse. It may be important for future research to study red tape in health care settings, especially in the ICU.

### 5.5 Leadership styles and organizational goals

Research on leadership lacks a clear concise definition.^112^, ^113^ Many studies have defined leadership using various dimensions, such as personality characteristics of the leader, a form of persuasion, and a power relation, among many others.^112^ Although leadership is a vague factor, some aspects are important to discuss when leading an ICU or ward. In turn, these aspects may also play a role in personal leadership, such as in taking charge in one's career. Although it is vague, some previous research has shown that some leadership styles may impact organizational performance^114^ but are highly context‐dependent.^114^ There are several approaches to studying and explaining leadership.^113^ Next, some initial insights relevant to the health care setting are provided.

According to Barrow,^112^ leadership has roughly three dimensions: leadership behavior factors, leader characteristics, and environmental factors. An important note is that the model is old and may not entirely fit the today's management context. It may still help to introduce leadership as a concept in the context of health care. *Leadership behavior* contains the actual behavior of a leader (or manager). It includes a focus on performing the job, and provide support where needed to achieve the goals of the department. This may also entail the ability to lead using more directive techniques.^112^

*Leader characteristics* also concerns the personality of the leader. These characteristics include technical expertise while at the same time understanding and manage the underlying organizational structure.^112^ Till recently, these characteristics were focused on masculine features of a leader. In today's economy, female leadership brings other along other dimensions that are important as a leader. For this reason, demographic‐physical characteristics may be a good addition to the model, but should not be solely based on masculine factors. Especially, in the light of diversity and inclusion, where including the background of the individual leader may also help to reduce the old stigma of how leadership is perceived in current society.

Finally, environmental factors that influence leadership include understanding the characteristics of the organization, team and group, and external stakeholders to the department and organization.^114^ Clearly, a leader should be aware of their own behavior, the interplay between the person and their environment, and between the departments in the organization and external stakeholders (health insurance companies, governments, etc.). Such a view of leadership is referred to as servant leadership.^115^ It is advised to adjust the leadership style to the characteristics of the employees, department, and organization to optimize the impact one may have. However, it is for future research to study servant leadership in the health care setting.

### 5.6 Objective and subjective performance

When at work people are supposed to perform their duties, though quantification of performance is usually very difficult. At the ICU, doing what is necessary to treat the patients is usually the responsibility of the team treating them. However, how would one define performance in the ICU? As with any performance indicator, it has benefits and drawbacks. Part of economic theory is concerned with perverse incentives.^116^ Such incentives may be exemplified by introducing financial incentives for researchers to publish that may not lead to actual performance. For instance, a recent study^116^ found that several countries that implemented cash incentives for scientists to submit papers to high‐end journals, ignoring the fact that there is a low probability of success. In a follow‐up study researchers found that the number of submission increased significantly while the number of resulting publication was stable.^116^, ^117^

In health care, one may focus on more objective performance measures, such as treating more patients per unit time. However, this may perversely lead to admission decisions where patients who are less severely ill will be admitted sooner as compared to more severely ill patients; in that case, these patients will recover sooner so that they can be discharged faster to the normal ward. Or, it may lead to only admitting the most severely ill (eg, highest APACHE IV score at admission), as these patients will usually die faster.

A focus of performance may also be on the quality of care delivered. A question remains on how to define what a good quality of care is. Frequently, quality of care is assumed to be the perceived care the patient received during admission. However, one may doubt whether the quality of care can be judged by the individual patient (who did not receive medical training). At the same time, complex treatment decisions can never be qualitatively evaluated by patients. While, in the end, one may argue it is this complex treatment decision that makes the quality of care excellent. From a patients' perspective, friendliness and communication skills are much more commonly evaluated rather than the actual treatment decisions.

At the ICU, there are options for comparing the performance among ICUs within a country,^118^ or even comparisons across countries.^118^, ^119^ The problem to date is that these comparisons are done once a year and cannot be used to direct steer a department more frequently than yearly and should be adjusted by the case mix of patients.

One may also focus on subjective performance; how is a colleague part of the team? One may perceive a colleague as a true team player when he steps in when a colleague is sick and takes over the shift. However, these measures are usually unconscious and difficult to quantify. However, these subjective performance measures may contribute to understanding how well a colleague is functioning in a team.

The present discussion about performance is not conclusive but merely a discussion about how difficult objective and subjective performance is. These discussions should also take into account possible *perverse incentives* and possible solutions to deal with them. Though little evidence has been provided by research attempting to associate individual and interpersonal factors with objective and subjective performance provided little evidence.^120^

## 6 EXTERNAL FACTORS: AGING POPULATION, LIMITED STAFF, AND TECHNOLOGICAL DEVELOPMENTS

There are three major external factors that play a role in designing HR policies in health care. These were identified as external, as they are external to the health care organization that puts pressure on delivering high‐quality care.

First, aging of the population increases pressure in two ways for an economy and many organizations. When more staff members retire and there is a limited supply of new employees on the labor market. This in turn puts pressure on the remaining workforce to perform the (same) job. Second, an aging population require more health care and thus it increases the demand for health care. Within the health care setting a smaller workforce and increasing health care demand require organizations to optimize available resources to perform the job.

Both the aging population, increasing workload together with limited staff members put pressure on digitization and automation of care processes. As mentioned above, telemedicine and distant monitoring of ICU patients^70^ as well as the use of big data^121^ may help to support the lack of personnel in the coming years or at least may decrease the gap between health care demand and supply.

## 7 SYNOPSIS: A STARTING POINT FOR FUTURE RESEARCH

HRM is a relevant and important topic for the ICU. Important factors are presented in this pragmatic review, which may serve as a starting point for future research. Currently, after the acute phase of the COVID‐19 pandemic, an aging population, and during a time of limited availability of staff members, the human capital of the organization is becoming increasingly important. Both management and research at the ICU may make use of incorporating existing theories and frameworks to study this in their own departments. Aligning the interests of the individual staff members with department and organizational goals is key in designing HR practices. Also, HRM practices impact at several levels of the organization (eg, individual, department, and organizational level). This may require a multilevel approach to studying HRM within the intensive care (see also Ref. 35). In addition, intervention studies (eg, by using a stepped‐wedge study design) may be needed to test whether effects of changes in HRM practices may lead to promising and measurable results. This with the goal to establish a learning health system.

Topics were presented related to the individual working in health care. Conscientiousness is one of the Big Five personality traits related to job performance. In addition, empathy and interpersonal mental processes are important factors to discuss. These so‐called “soft skills” are immensely important when interacting with colleagues and patients. As a result, these factors will lead to higher quality of care and patient perception of safety.

Further, as discussed earlier, the relationship between job demands and resources, work engagement and performance is central theme in HRM. It may play an important role for managers and department chairs in how their HR policies are related to performance. Also, red tape plays an important role in understanding the bureaucratic issues inhibiting work engagement. For example, nurses complain about the workload caused by administrative duties and not being able to care for their patients. It is important to note that studying the relationship between work engagement and performance within the ICU should therefore be accompanied by also examining red tape, perceived safety, and/or the other HR factors described in this pragmatic review. The key is to keep employees involved and engaged with their work through proper support to enhance job motivation.^42^

At the department and organizational level, the purpose is to set and align department and organizational goals with the interest and qualities of individual staff members. This requires a helicopter view of the performance goals of the organization and department, combined with insights into the qualities of the personnel. A strategic HRM plan should be established for this purpose helping the leadership of a department steer its performance. Such a plan not only provides goals for the department but also aims to provide a direction for what type of personnel should be hired.

## 8 CONCLUSION

This pragmatic review presented an overview of current and important topics in the field of HR. The topics discussed are a nonexhaustive list of topics but highlight those that are particularly relevant for the ICU and health care in general. In addition, it may guide future research agendas, and for managers, it may guide understanding the behavior of individual staff members.

## AUTHOR CONTRIBUTIONS

All of the authors wrote, read, and approved the content of the study.

## FUNDING INFORMATION

This work was supported by the ZonMW (Grant number: 10430132220026).

## CONFLICT OF INTEREST STATEMENT

The authors do not have any conflicts of interest to declare.
